# Identification of basement membrane-related signatures for estimating prognosis, immune infiltration landscape and drug candidates in pancreatic adenocarcinoma

**DOI:** 10.7150/jca.89665

**Published:** 2024-01-01

**Authors:** Feng Zhou, Yang Liu, Dingwei Liu, Yong Xie, Xiaojiang Zhou

**Affiliations:** 1 Department of Gastroenterology, Digestive disease Hospital, The First Affiliated Hospital of Nanchang University, Nanchang, Jiangxi Province, China.; 2 Gastroenterology Institute of Jiangxi Province, Nanchang, Jiangxi Province, China.; 3 Key Laboratory of Digestive Diseases of Jiangxi Province, Nanchang, Jiangxi Province, China.; 4 Jiangxi Clinical Research Center for Gastroenterology, Nanchang, Jiangxi Province, China.

**Keywords:** Pancreatic adenocarcinoma, Prognosis, Immune infiltration, Drug, Basement membrane

## Abstract

**Background:** Pancreatic adenocarcinoma (PAAD) is a frequent digestive system cancer, which has high mortality and bad outcome. However, the role of basement membrane (BM)-related gene in assessing patient's outcome, microenvironment and treatment response remain unclear.

**Methods:** Basement membrane (BM)-associated genes were detected by univariate and least absolute shrinkage and selection operator (LASSO) Cox regression analyses using data from the TCGA databases. A risk score system was constructed to distinguish patients in the high- and low-risk groups. Prognostic gene distribution in various immune cell forms was explored through scRNA-seq. Immune cell infiltration was assessed using CIBERSORT and ESTIMATE. The IC50 of common chemotherapeutic drugs and useful molecule compounds were evaluated. The mRNA and protein expression of important signatures were validated utilizing GEPIA and HPA databases.

**Results:** Compared to low risk PAAD patients, PAAD patients with high risk showed a significant much worse overall survival (OS). Risk score of BM-associated genes could estimate patient outcome well, and areas under the curve (AUC) of receiver operating characteristic (ROC) survival curve were 0.76, 0.85, and 0.85 at 1-, 3-, and 5-year. Clinical impact curve (CIC) curve demonstrated the clinical importance of risk score. scRNA-seq revealed that BM-related genes were mainly distributed in malignant cells. Significant variations existed in the immune microenvironment, immune checkpoint expression and chemotherapy response between the studied groups. Furthermore, the mRNA expression levels of FAM83A, LY6D, MET, MUC16, MYEOV, and WNT7A were elevated in PAAD tissues, while the protein expression patterns of LY6D, MET, MUC16, and WNT7A were higher in tumor sample. RO-90-7501, Scriptaid, TG-101348, XMD-892, and XMD-1150 may be valuable small molecule drugs to treat PAAD.

**Conclusions:** In conclusion, we develop a novel BM-related gene signature provide new insights and targets for the diagnosis, outcome estimation, candidate drugs and therapy management of PAAD patients.

## Introduction

Pancreatic Adenocarcinoma (PAAD), a highly fatal malignancy, has a rate of less than 10% for 5-year survival 1. It was predicted that PAAD will become the second main reason of cancer mortality by 2030^2^, ^3^. At present, surgical treatment combined with adjuvant chemotherapy is still a standard treatment, but PAAD cannot be effectively screened due to the hidden anatomic location, insidious characteristics, lack of biomarkers, and many patients have advanced disease and no good treatment is available^3^. For high-risk patients with poor prognosis, individualized treatment strategies may help improve survival and quality of life. Subsequently, reliable predictive and novel markers are urgently required to classify patients and optimize clinical decision-making accurately.

Basement membranes (BMs) belong to the extracellular matrix (ECM) and surround most tissues^4^. The main components of BM can be divided into collagen, laminin and fibronectin. To engage in metastasis, tumor cells must infiltrate through BMs. Loss of BM-associated protein expression is among the highest critical reasons of cancer^5^. In a way independent of matrix metalloproteinase, cancer-associated fibroblasts (CAFs) enhance cancer cell invasion by BM^6^. Recent research indicates that the epithelial-mesenchymal transition (EMT) vastly depends on the BM and BM can be utilized to design cancer therapeutics to target metastasis^7^. Therefore, BM is critical for tumor metastasis and progression.

Few BM-related biomarkers are good predictors of the PAAD patient prognosis. Fortunately, a high-quality article published in May 2022 in the journal of *SCIENCE ADVANCES* found that a network of homologous human and animal proteins located in the BM by comprehensive analysis^8^. At present, there is no comprehensive analysis report on the prognostic value of BM-related proteins in PAAD patients. Identifying potential prognostic markers associated with treatment benefit can help personalize treatment for patients with PAAD. Therefore, finding markers is crucial to predict the outcome and treatment effect of PAAD. This research aimed to detect the key BM-related biomarkers for PAAD patients by The Cancer Genome Atlas (TCGA). Accordingly, our research can provide certain guiding value for the basic research in the field of cancer, especially PAAD.

## Materials and Methods

### Data collection

**Figure 1:**
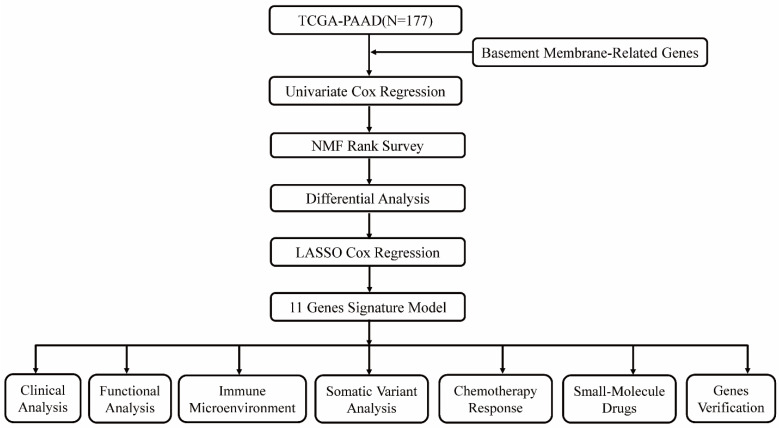
The flowchart of this study.

Various clinical data comprising transcriptome expression, gene mutation profiles, and transcriptome expression profiles of PAAD patients were retrieved from TCGA database. Those patients whose survival data was incomplete were excluded. Eventually, 177 PAAD patients were enrolled in the TCGA-PAAD cohort. Two hundred and twenty-four BM-related genes were obtained from a recent literature report^8^. Forty-two survival related BM genes were obtained by survival analysis in the TCGA-PAAD dataset. Then, two clusters were confirmed as optimal by indicators of NMF rank survey, and cluster 2 was found to have a poor prognosis. In order to analyze the reasons for the poor prognosis, differential genes between the two clusters were compared, and 587 DEGs were analyzed. Modeling analysis of these differential genes was performed in the training set. Moreover, univariate analysis found that 65 of the differential genes were BM genes associated with survival. Following lasso analysis, 11 genes were identified to generate the risk score, and the median risk score was employed to split the high and low risk groups. Finally, nine genes were chosen for verification analysis. Figure 1 shows the analysis process diagram of this research.

### Identification of consensus clustering via NMF

The nonnegative matrix factorization (NMF) clustering method was employed to cluster PAAD patients. This process was performed by R package “NMF” with for typing with rank set to 2:10, choosing the standard “brunet”, and performing 50 iterations. Finally, determine the optimal clustering number (k=2). The idea of NMF: V=WH (W is the weight matrix, H is the characteristic matrix, V is the original matrix. W: basis, H: coefficients, Consensus: Consensus Clustering (Consistent clustering).

Silhouette is an approach for interpreting and validating the consistency of data clusters. The silhouette value quantifies the similarity of an object to its own cluster (cohesion) relative to other clusters (separation). The silhouette varies from 1 to +1, with a high value indicating that an object is well-suited to its own cluster but poorly matched to adjacent clusters. If the majority of items show a high value, the clustering configuration is suitable. If a significant proportion of data points have a negative or low score, the clustering configuration may have too few or too many clusters.

### Risk score of key prognostic signature

The TCGA-PAAD dataset were split into training and validation cohorts according to the ratio of 7:3. Survival analysis of training cohort obtained 65 survival genes (*P*<0.01), eleven genes were eventually obtained using Lasso regression, and the model was created. Afterward, risk score of each PAAD patient was measured. This equation was employed to measure the risk score: Risk Score=ΣA*B. (A: expression of every gene, and B: corresponding coefficient). Patients with PAAD were categorized into high- and low-risk groups based on the appropriate risk score cutoff value. R packages "survival" and "survminer" were employed to evaluate the survival rates of patients in the two risk groups using Kaplan-Meier (KM) curves. R package "timeROC" was employed to generate receiver operating characteristic (ROC) curves. ICGC data set was utilized to further verify the correlation between differential genes and survival in the high-low risk group. Subgroup analyses of clinically relevant factors (sex, stage, and age) were conducted to assess the function of BM-associated genes on PAAD patient outcome.

### Assessment of prognostic value

After further excluding patients for whom clinical information was lacking, univariate and multivariate Cox regression analyses were performed to evaluate the individual prognostic significance of risk scores, age, sex, and stage in pancreatic adenocarcinoma (Supplementary Table 1). ROC curves were employed to gauge the predictive accuracy of risk scores at 1, 3, and 5 years, quantified by the area under the ROC curve (AUC). Additionally, we employed the clinical impact curve (CIC) to assess the practical utility of our risk score model. This allowed us to measure the net benefit of using the risk score to guide clinical decisions, factoring in threshold probabilities for clinical interventions.

### Enrichment analyses of DEGs

Using the "limma" R package and volcano plots, the DEGs between the two risk groups were examined. GeneMANIA (http://www.genemania.org) is an adaptable, user-friendly web-based application for creating gene function hypotheses, assessing gene records, and selecting genes for functional tests 9. The PPI networks of key prognostic genes were predicted using GeneMANIA. Metascape studied the functional and pathway enrichment analyses of DEGs.

### scRNA-seq analysis

The Tumor Immune Single-cell Hub (TISCH) database (http://tisch.comp-genomics.org/) comprises 79 high-quality single-cell transcriptome datasets of 27 tumors with matching clinical data, which can enable thorough single-cell level cell type annotation. This database offers comprehensive data, simple operation, user-friendliness, and data visualization^10^. The Uniform Manifold Approximation and Projection (UMAP) plot was employed to depict the dispersion and expression of predictive-associated genes in the PAAD sample according to the TISCH database.

### Immune microenvironment analysis

Employing CIBERSORT method, the infiltrating abundance of 22 kinds of immune cells in high- and low-risk patients was determined. Each PAAD patient's stromal, immunological, and ESTIMATE scores and tumor purity were detected utilizing the ESTIMATE method. Moreover, MHC molecules expression and 36 frequent immune checkpoints among the various risk groups were compared.

### Somatic mutation analysis

Somatic mutation profiles from TCGA database were collected, and then the "maftools" R tool was employed to create a waterfall plot to show The rate of somatic mutations and the distribution of variable gene forms in the two studied groups. Furthermore, to examine the underlying molecular pathway of developing PAAD, the mutually exclusive and co-occurrence of mutated genes were examined between two risk groups.

### Chemotherapy sensitivity and potential medications

The Genomics of Drug Sensitivity in Cancer (GDSC) Project (https://www.cancerrxgene.org/) is an accessible to the public genomics database of antitumor treatment response that identifies the molecular characteristics of tumor and predicts the target response to antitumor medicines. Sensitivity of PAAD patients to common chemotherapy drugs was predicted by GDSC database. Calculation of the half maximal inhibitory concentration (IC50) for frequent chemotherapeutic drug in PAAD patients by utilizing R tool "pRRophetic". Connectivity Map (CMap) database (http://www.broadinstitute.org), a biological database, uncovers the functional connections between small molecule drugs, genes, and disease conditions^11^, ^12^ . Upregulated and downregulated DEGs between the two studied groups were uploaded to the CMap database to forecast small molecule medicines that could be utilized to treat PAAD (The enrichment scores ranged from -1:0).

### Gene verification

The GEPIA website (http://gepia2.cancer-pku.cn/) was employed to examine the variations in mRNA expression and overall survival between cancer and healthy individuals. Additionally, we used the HPA website (https://www.proteinatlas.org/) to analyze the protein expression of key genes in human cancer and healthy tissues. Protein expression score was detected using immunohistochemical (IHC) staining intensity and staining cell proportion.

### Statistical analysis

R program (v4.0.5) and related R tools were utilized to conduct all graphs and statistical analyses. Variation between two studied groups were examined utilizing t- test. Wilcoxon test was utilized to study the nonparametric comparisons between two groups. Utilizing log-rank test, survival was conducted.

Using Spearman correlation analysis, the association between two continuous variables was evaluated. P < 0.05 was judged statistically significant.

## Results

### Identifying BM-associated molecular subtypes

**Figure 2:**
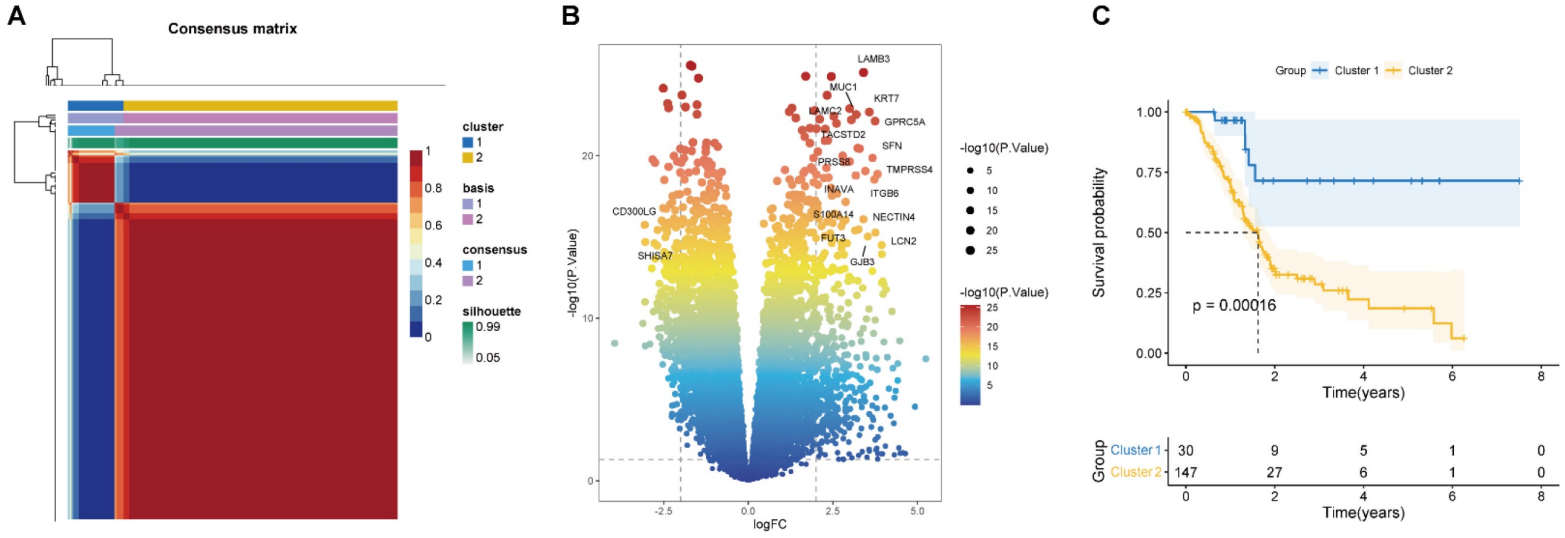
** (A)** Consensus map of NMF clustering (k=2). The idea of NMF: V=WH (W is the weight matrix, H is the characteristic matrix, V is the original matrix. W: basis, H: coefficients, Consensus: Consensus Clustering (Consistent clustering). **(B)** Volcano map of DEGs in cluster 1 and 2. **(C)** Survival curve of two clusters.

According to the 224 BM-associated genes, data of 177 PAAD patients were retrieved from TCGA database. We gained forty-two survival-associated genes using Cox regression analysis. Next, two molecular subtypes of PAAD were identified by the NMF algorithm. Finally, the clusters 1 and 2 were obtained by BM-associated genes under NMF clustering (Figures 2A and S1). DEGs of the two clusters were compared by volcanic map (Figure 2B). According to survival analysis, the prognosis of cluster 1 was significantly better than cluster 2 (Figure 2C).

### Identification and validation of the predictive signature

**Figure 3:**
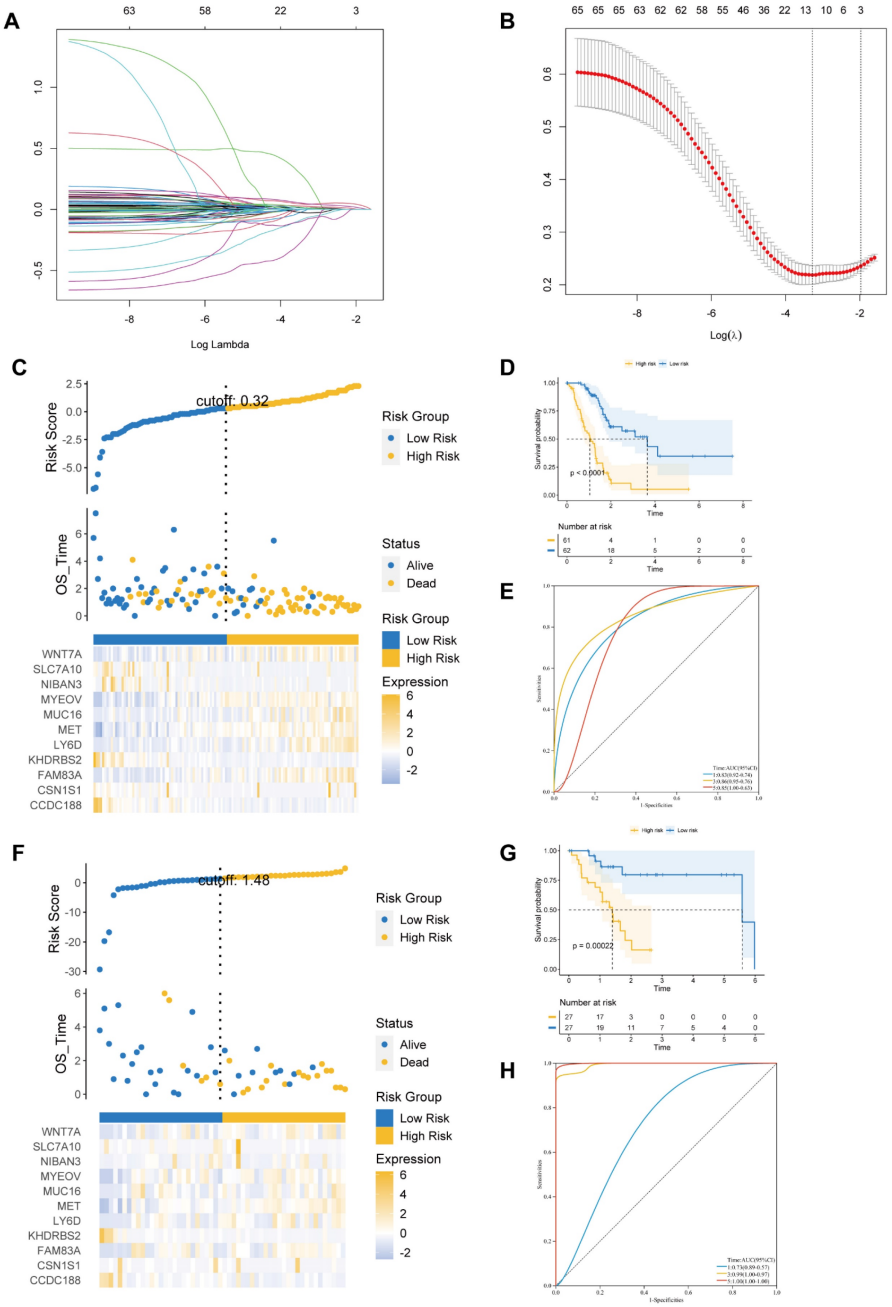
** (A)** The LASSO coefficient profile of 11 basement membrane-related prognostic signatures.** (B)** Identification of the optimal tuning parameter (log λ) LASSO model using cross-validation. **(C, F)** The distribution map of risk score (upper), survival time (middle), and heatmap for the expression of eleven genes (below) in the training cohort** (C)** and validation cohort **(F). (D, G)** Survival curve of the high- and low-risk groups in the training cohort** (D)** and validation cohort **(G). (E, H)** ROC curve of the basement membrane-related prognostic gene for predicting the 1-, 3- and 5-year OS of PAAD patients in the training cohort **(E)** and validation cohort**(H).**

We identified 587 DEGs between cluster 2 and cluster 1. Next, LASSO Cox regression was employed to examine eleven prognostic genes related to BM according to DEG (Figures 3A and B). There were 123 PAAD patients in training and 54 patients in validation cohorts at a 7:3 ratio. The estimated risk scores were segmented into high- and low-risk groups in accordance with the median risk scores (Risk Score=ΣA*B. (A: expression of each gene, and B: corresponding coefficient). Figure 3C shows the survival times for PAAD patients. A total of 11 key genes expression in the two risk groups were depicted by heatmap (Figure 3C). In addition, the survival curve revealed a reduced survival rate in high- than the low-risk group (Figure 3D). Above all, the areas under curves (AUCs) were 0.83, 0.86, and 0.85 at 1-, 3-, and 5-year survival, respectively, suggesting that BM-related prognostic genes could be useful in forecasting PAAD patients' outcome (Figure 3E).

We further performed similar analyses to detect the significance of prognostic genes in validation and total cohorts. The risk score was measured using the exact equation. Figure 3F and Supplementary Figure S2A shown the risk score, survival time, and key genes' expression and distribution. From figures 3G and S2B, we can conclude that the prognosis was significantly increased in the low- compared to high-risk group in both the validation and the total cohorts. AUC at 1-, 3-, and 5-year of validation cohort were 0.73, 0.99, and 1.00, of total cohort were 0.76, 0.85, and 0.85, respectively (Figures 3H and S2C). At the same time, we observed that the prognosis was significantly better in the low- compared to high-risk group in the ICGC dataset. AUC at 1- and 3-year of ICGC cohort were 0.71 and 0.84 (Figure S3).

### Functional analysis

**Figure 4:**
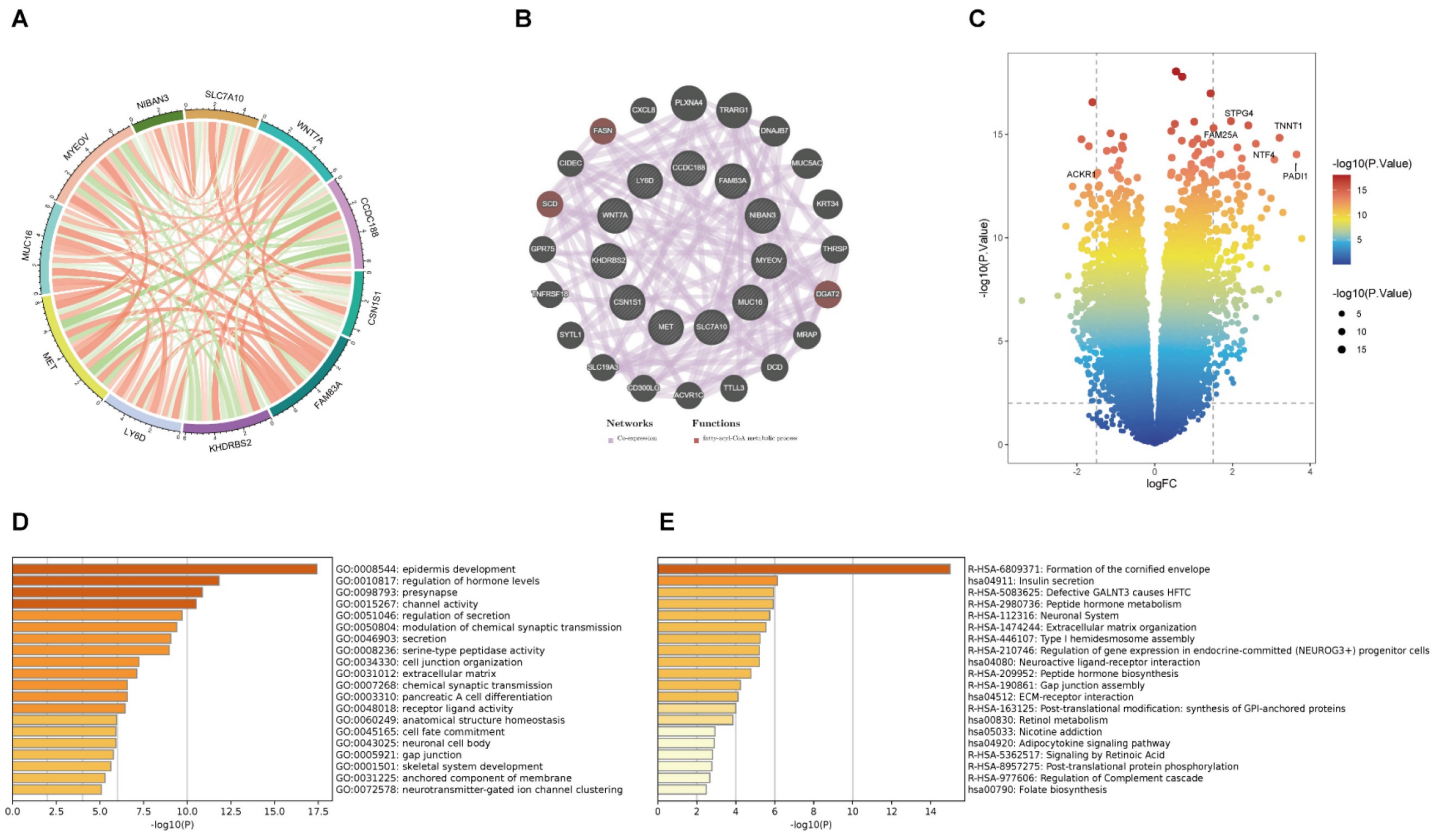
** (A)** The correlation analysis between eleven key prognostic genes. **(B)** Coexpression PPI network of basement membrane-related genes by the GeneMANIA database. **(C)** Volcano plot of DEGs in the high- and low-risk groups.** (D)** GO analysis of DEGs.** (E)** KEGG analysis of DEGs.

This is the correlation analysis among 11 BM-related genes (Figure 4A). In addition, eleven gene PPI networks were predicted through the GeneMANIA website (Figure 4B). Findings show that MUC16 and LY6D are positively correlated, while there is a negative correlation between CCDC188 and MET. Next, we developed the interaction network of 11 key prognostic genes by GeneMANIA website. The function of SCD, FASH, and DGAT2 was mainly related to the fatty-acyl-CoA metabolic process. Further, we modeled the differential genes obtained in Figure 2B to obtain risk scores (categorized into high and low risk groups by the median risk score). In total, 352 differential genes between the high and low risk groups were used for enrichment analysis. The volcanic map of DEGs between two risk groups (Figure 4C). The volcano plot revealed 352 DEGs between two risk groups, including 161 downregulated genes and 191 upregulated genes. The DEGs were principally enriched in “epidermis development”, “regulation of hormone levels”, “presynapse”, “channel activity”, and “regulation of secretion” based on GO analysis (Figures 4D and S4A). KEGG analysis shown that the DEGs were principally enriched in “Formation of the cornified envelop”, “Insulin secretion”, “Defective GALNT3 causes HFTC”, “Peptide hormone metabolism”, and “Neuronal System” (Figures 4E and S4B).

### Clinicopathological manifestations

**Figure 5:**
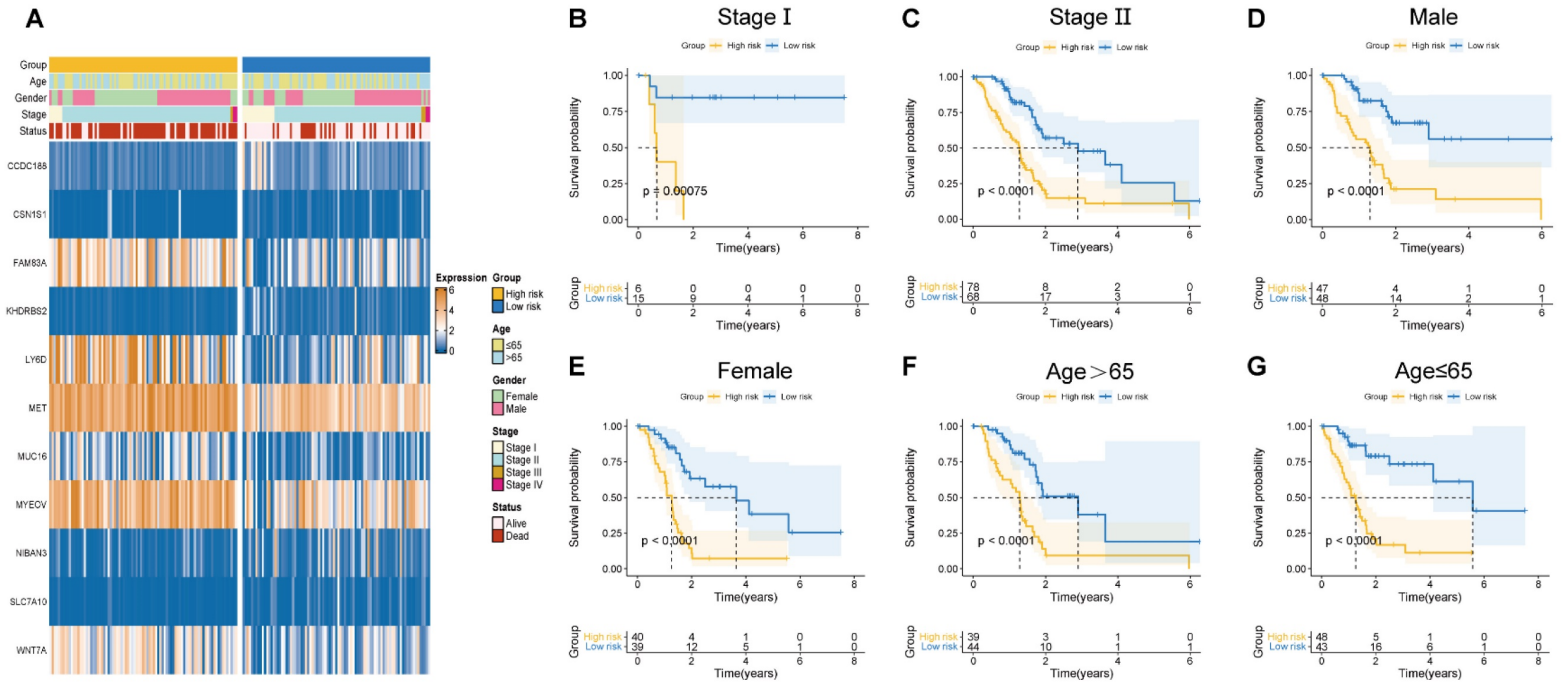
** (A)** Correlation of basement membrane-related genes with clinicopathological manifestations.** (B-G)** Survival probability of patients in different risk groups stratified by stage** (B, C)**, gender **(D, E)**, and age **(F, G)**.

Herein, the risk scores of eleven BM-related key signatures were calculated for each PAAD patient. We examined the association between the BM-associated genes expression and clinicopathological features using heatmap (Figure 5A). Findings revealed that BM-associated genes expression was significantly related to sex, survival status, stage, and age. After that, we carried out a survival analysis of PAAD patients categorized by sex, age, as well as stage. According to our findings, survival time was significantly reduced in patients in the high- compared to those in the low-risk group (Figures 5B-G). Our results show that there is only a difference between the risk score of stages I and II. Figure S5A shows non-significant variation between the risk score for stage II, III, and IV. Additionally, a non-significant variation existed in risk score between subgroups of age and gender (Figure S5B, C). In summary, the BM-related signature could be promising the predictive treatment of PAAD patients.

### Prognostic factors and risk scores

**Figure 6:**
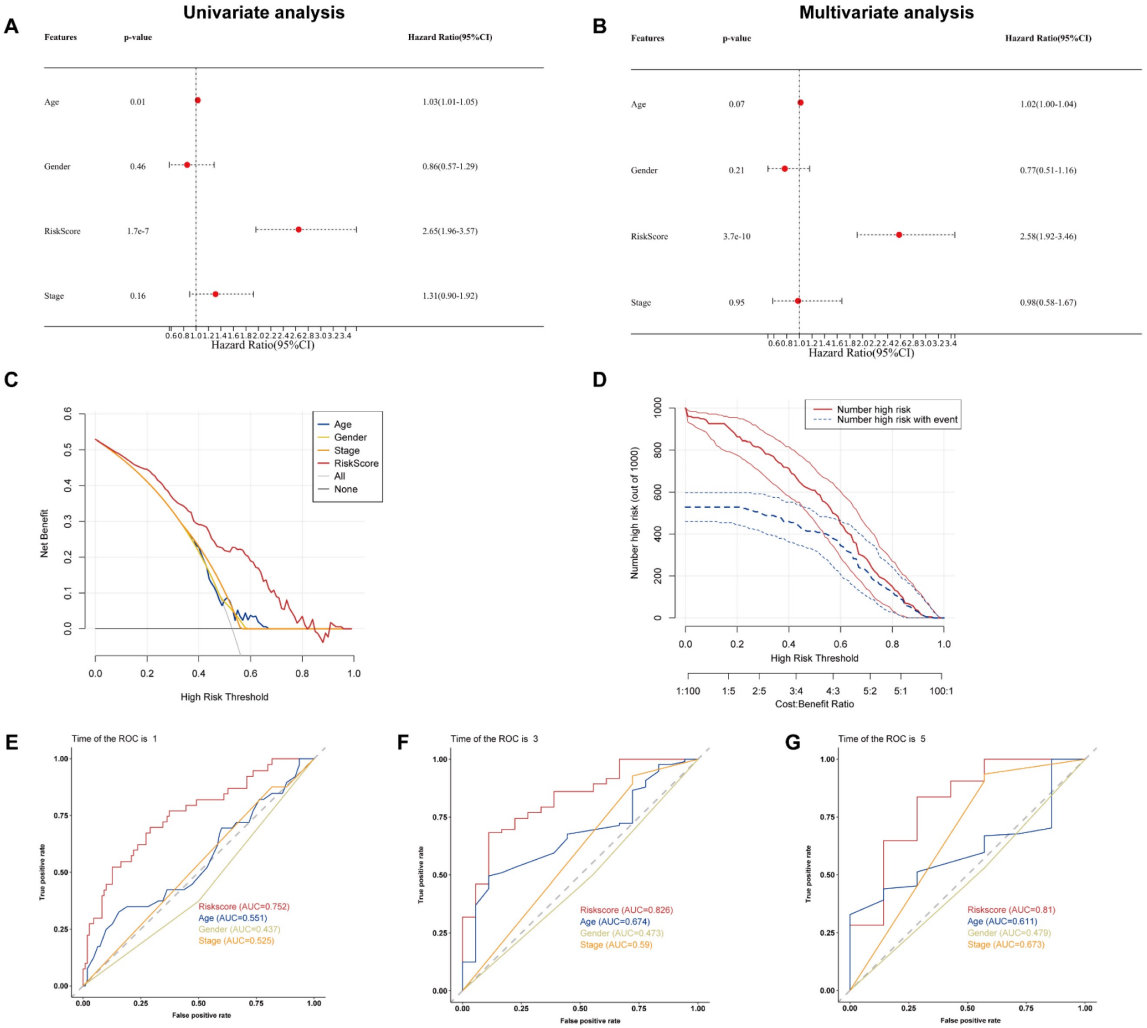
** (A)** Univariate and **(B)** multivariate Cox regression analysis of the basement membrane-related genes. **(C)** DCA and **(D)** CIC of the model. **(E-G)** ROC curves of the model for predicting the 1-, 3- and 5-year OS.

To examine if the BM-associated signature is an independent predictive variable for PAAD, we conducted cox regression analyses on the risk score and clinical data. Independent variable: age, sex, risk score, and stage. Dependent variable: survival data (including binary outcome variable and continuous survival time variable). The present findings demonstrated that the hazard ratio (HR) levels of risk score were 2.65 (95% CI: 1.96-3,57) and 2.58 (95% CI: 1.92-3.46) in univariate and multivariate Cox regression analyses, respectively (Figures 6A and B). Particularly, HR demonstrated an raising trend from univariate to multivariate Cox regression analysis. We found that age, gender, and stage had minimally affect the outcome (P>0.05), whereas only risk score had a significant effect (*P*<0.05). Therefore, we did not construct nomogram. By comparing the predictive value of risk score sex, age, and stage, it was discovered that risk score may anticipate the outcome well. Results show that the DCA and CIC curve suggested that the risk score may acquire the highest net gain relative to other clinical variables, showing excellent validity and reliability (Figures 6C and D). Furthermore, we evaluated the predictive ability of the risk score to other clinical characteristics. Compared to other clinical characteristics, the risk score for estimating 1-, 3-, and 5-years OS had the greatest AUC of 0.752, 0.82, and 0.81, respectively (Figures 6E-G). According to these data, the risk score could effectively anticipate the outcome of PAAD patients and assist in the discovery of therapeutic treatment methods.

### Association of BM-associated signature with single cell properties

**Figure 7:**
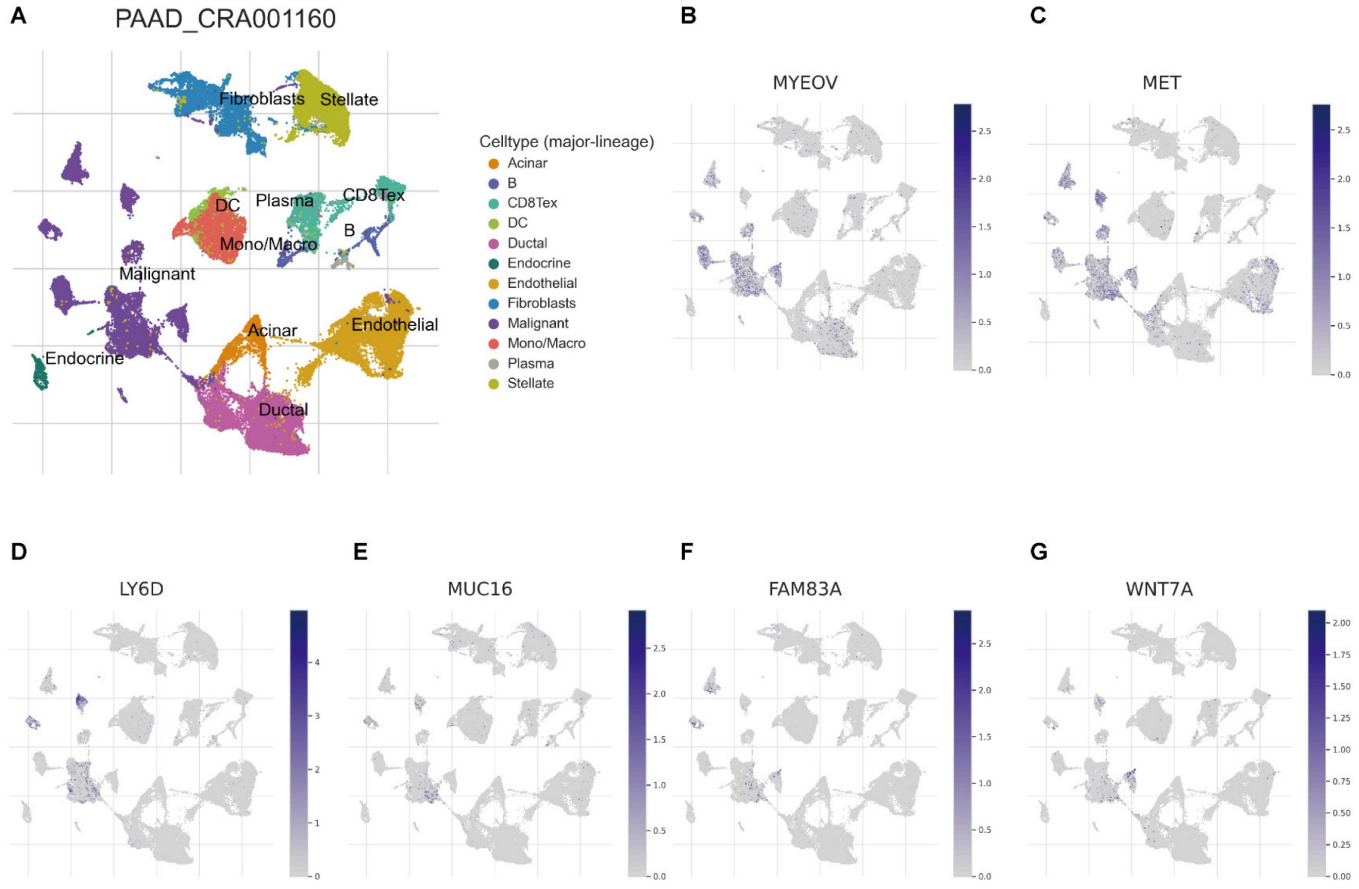
** (A)** UMAP plot of twelve major cell clusters in the PAAD tumor microenvironment. The distribution of MYEOV **(B)**, MET **(C)**, LY6D **(D)**, MUC16 **(E)**, FAM83A **(F)**, WNT7A **(G)** in the cell subsets.

Recently, developing scRNA-seq has evolved into a crucial method for revealing variations across cell populations and characterizing diverse cell populations. To evaluate the involvement of BM-associated gene in the tumor microenvironment (TME), therefore, this research evaluated the scRNA-seq data of PAAD using the TISCH database. UMAP plot depicts cell clusters, all of which are annotated according to its unique signature genes (Figure 7A). The majority consisted of acinar, CD8+, DC, ductal, endocrine, endothelial, malignant cells, and monocytes/macrophages. Moreover, B, plasma, mast cells, and fibroblasts are essential immune microenvironment constituents. Also, we evaluated the distribution of MYEOV, MET, LY6D, MUC16, FAM83A and WNT7A in 12 cell clusters (Figures 7B-G). According to our findings, these genes were primarly distributed in malignant cells. But, CCDC188, KHDRBS2, and SLC7A10 were rarely found in malignant cells (Figure S6). Overall, our results showed a clear correlation between BM-related important signatures and PAAD tumor microenvironment, and the signature is potentially act as a marker for anticipating the effectiveness of treatment in PAAD patients. In addition, there are also significant differences in BM-associated signature among different cancer types (Supplementary Table 2).

### Association between BM-associated signature and immune microenvironment

**Figure 8:**
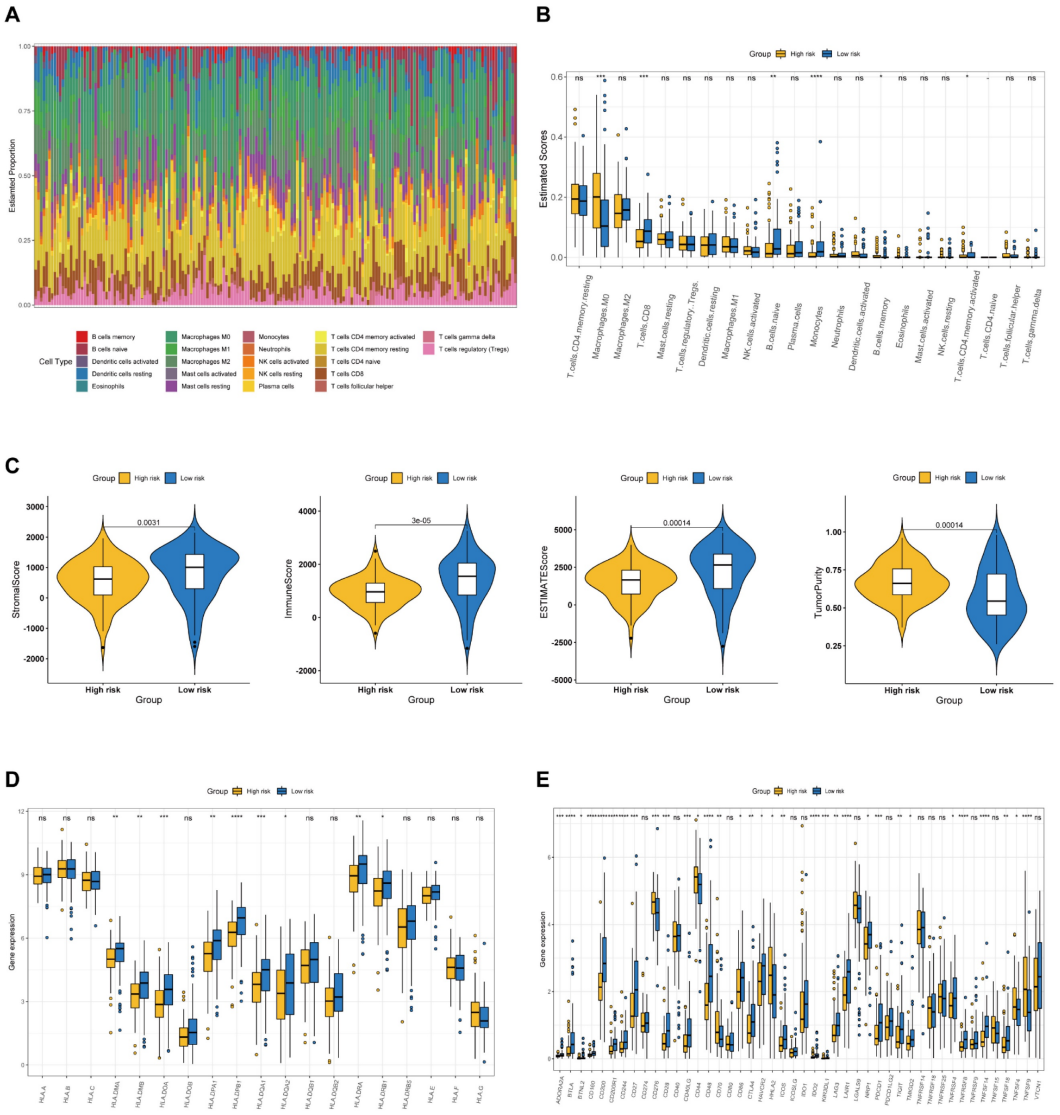
** (A)** The estimated proportion of immune cells in each tumor sample. **(B)** The ESTIMATE score of different immune cell subtypes in different risk groups. **(C)** The stromal score, immune score, ESTIMATE score, and tumor purity in the high- and low-risk groups. **(D)** The expression of MHC molecules in different risk groups. **(E)** The expression of immune checkpoint related genes in different risk groups.

We examined the immunological landscape in the TCGA-PAAD dataset utilizing the CIBERSORT method to examine the association between the BM-associated signature and the immune microenvironment. In total, 22 immune cells were represented by a stacked bar plot, as illustrated in Figure 8A. We discovered that macrophages and CD4+ T cells comprised most of all immune cells. Then, the proportional quantities of immune cells in distinct risk groups were determined. CD8+ T cell infiltration density was greater in the high-risk group (Figure 8B). After that, we examined the stromal, the immune, and the ESTIMATE scores as well as the tumor purity between risk groups. Findings indicate that the stromal, immunological, and ESTIMATE scores were significantly greater in low- compared to high-risk group (P < 0.05), although the tumor purity was greater in high-risk group (Figure 8C). The data presented above revealed significant changes in the immune cell microenvironment between the two studied groups, which may contribute to disparities in immune function between the two risk groups. In addition, we discovered that the majority of MHC molecules expression was greater in the low-risk group (Figure 8D). Immunotherapy, which can generate a more persistent response in cancer patients than traditional chemotherapy and gives hope for cancer treatment, has made considerable and quick advances recently. Consequently, 46 immune checkpoint genes expression was studied in individuals at high and low risk. Additionally, we discovered high expression of immune checkpoint genes CD276, CD44, CD70, HHLA2, TNFSF4 and TNFSF9 in the high-risk group, whereas the immune checkpoint genes CD27, CD28, CD40LG, CD48, CD86, CTLA4, HAVCR2, ADORA2A, NRP1, PDCD1, BTLA, BTNL2, CD160, ICOS, IDO2, KIR3DL1, LAG3, LAIR1, TIGIT, CD200, TNFRSF8, TNFSF14, CD200R1, CD244, TMIGD2, TNFRSF4, and TNFSF18 revealed high expression in the low-risk group (Figure 8E). Our analysis demonstrate that the BM-associated signature can possess potential clinical value in anticipating PAAD immunotherapy.

### Correlation between the BM-associated signature and mutation status

**Figure 9:**
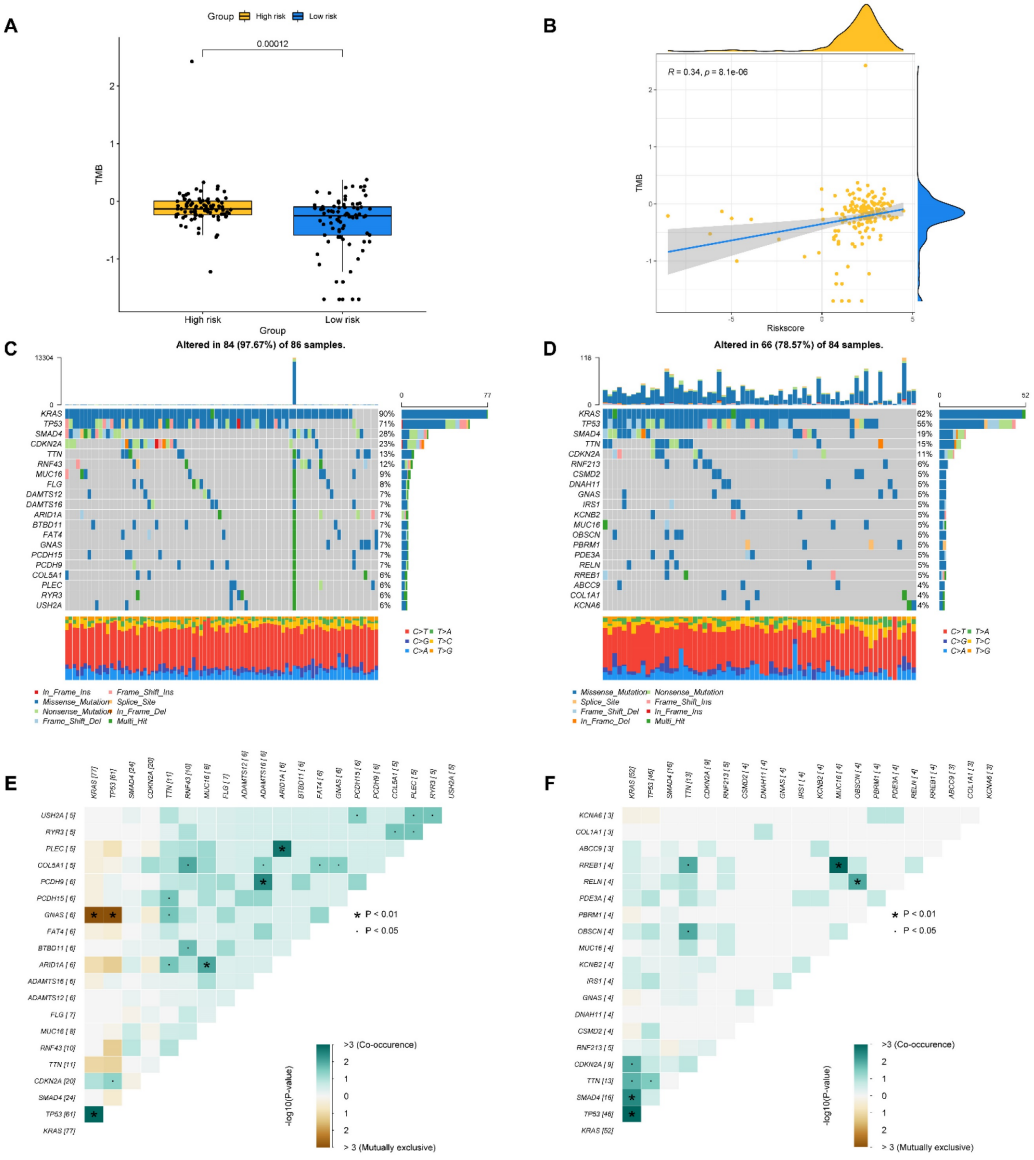
** (A)** The difference of TMB in different risk groups. **(B)** The relationship between TMB and riskscore. **(C, D)** The distribution of somatic mutation in the high-risk **(C)** and low-risk groups **(D)**. **(E, F)** The different mutations of genes in the high-risk **(E)** and low-risk groups **(F)**.

The tumor burden mutation (TMB) may have certain influence on the treatment of tumor, thus TMB analysis was performed for each PAAD patient. In Figure 9A, it is found that the TMB was significantly higher in high- compared to low-risk group. Moreover, we evaluated the latent relevance of risk score and TMB in PAAD patients. In addition, we investigated the potential link of TMB to risk score, and observed a positive relationship between them through correlation analysis (r = 0.34, P<0.05, Figure 9B). After that, we studied the influence of the BM-associated gene on somatic mutations in PAAD patients. The waterfall plots depict the mutational landscapes of high- and low-risk groups (Figures 9C and D). In the high-risk group, the highest five mutated genes were KRAS (90%), TP53 (71%), SMAD4 (28%), CDKN2A (23%), and TTN (13%). The highest five mutated genes were KRAS (62%), TP53 (55%), SMAD4 (19%), TTN (15%), and CDKN2A (11%) in the low-risk. Importantly, the frequency of TTN mutations was lower in the high- in comparison to the low-risk group. Additionally, missense mutations were the most prevalent kind of mutation in both risk subgroups. The cooccurrence and mutual exclusivity of the mutant genes in the two risk groups were then compared. In the high-risk group, mutated genes including KRAS and TP53 co-occurred (Figure 9C). The same thing happened in the low-risk group (Figure 9D). Mutual exclusivity of clearly mutated genes was clearly existed between GNAS and TP53, KRAS in the high-risk group (Figure 9E and 9F).

### Chemotherapy response and small molecule drug screening

**Figure 10:**
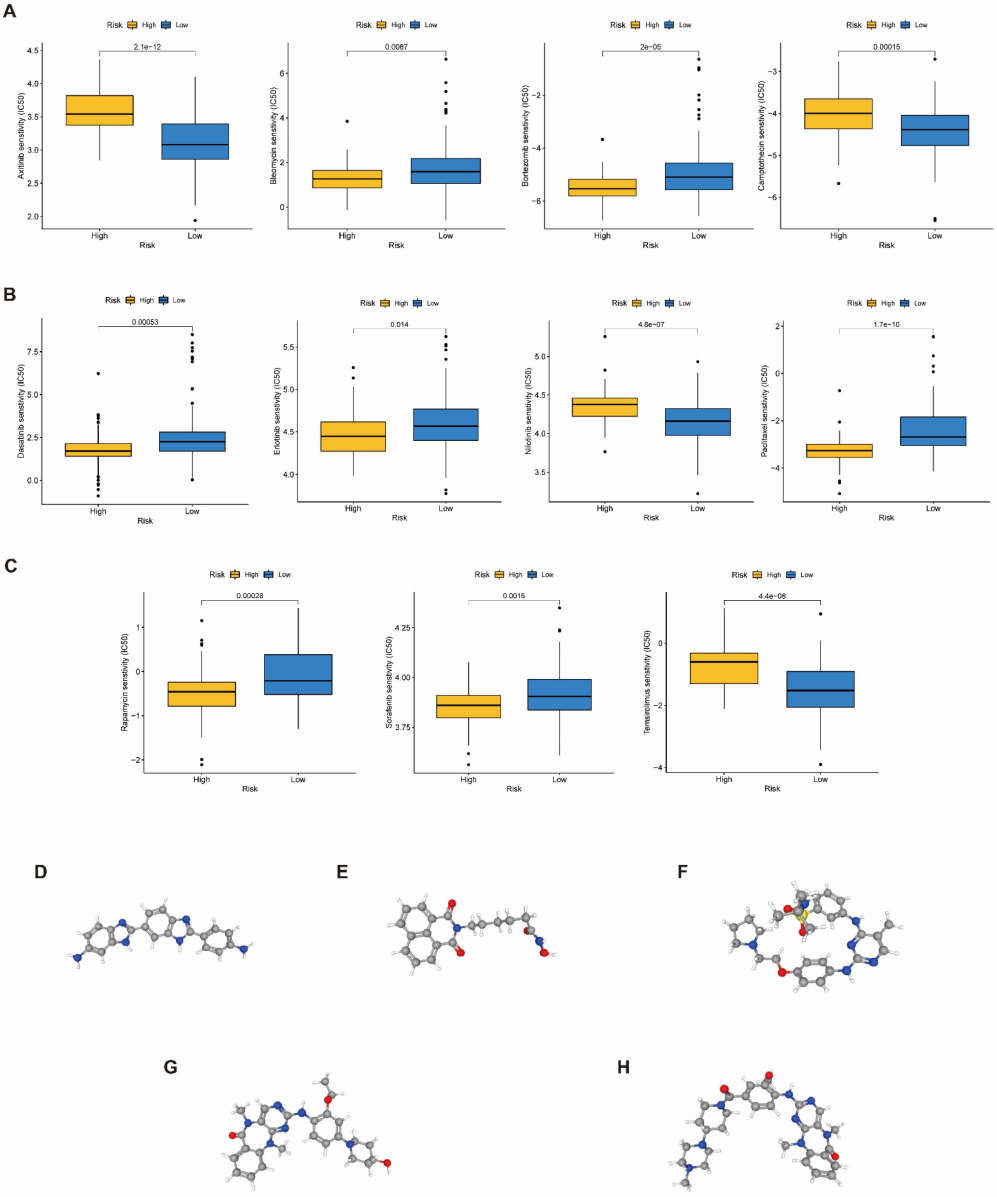
** (A-C)** The difference of chemotherapeutic drugs sensitivity in different risk groups. **(D-H)** The structure of potential drugs, including RO-90-7501 **(D)**, Scriptaid** (E)**, TG-101348 **(F)**, XMD-892 **(G)**, XMD-1150 **(H)**.

To raise the advantage of chemotherapy in PAAD patients, the prognostic capbaility of BM-associated signature was assessed for the effectiveness of typical chemotherapies in various risk patient. IC50 values for medications (axitinib, camptothecin, nilotinib, and temsirolimus) was significantly increased in high- compared to low-risk group (*P*<0.05, Figure 10A-C), which shown that these chemotherapeutic medications possess higher clinical effectiveness in high-risk patients. The current analysis findings revealed that the BM-associated signature has the ability to predict the chemotherapy effectiveness in PAAD patients. Furthermore, the DEGs between two risk groups (161 downregulated and 191 upregulated) were uploaded to the CMap database and anticipated five small molecule drugs that could be successful for PAAD therapy, namely, RO-90-7501 (Figure 10D), Scriptaid (Figure 10E), TG-101348 (Figure 10F), XMD-892 (Figure 10G), and XMD-1150 (Figure 10H).

### Gene expression

**Figure 11:**
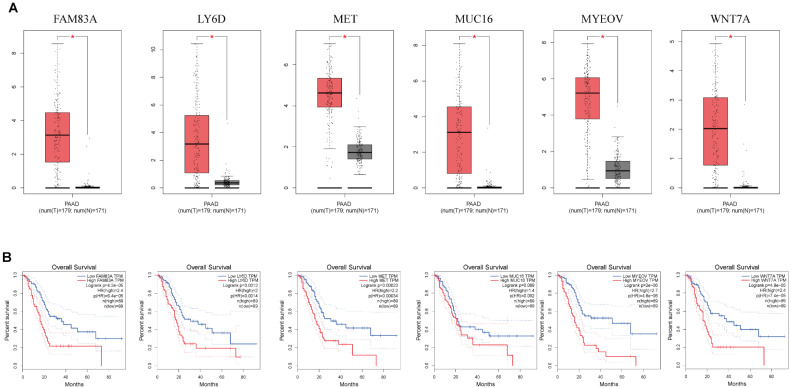
** (A)** The mRNA expression of key genes (FAM83A, LY6D, MET, MUC16, MYEOV, WNT7A) in PAAD and normal samples determined by GEPIA database. **(B)** Overall survival analysis of prognostic genes (FAM83A, LY6D, MET, MUC16, MYEOV, WNT7A) in PAAD determined by GEPIA database.

**Figure 12:**
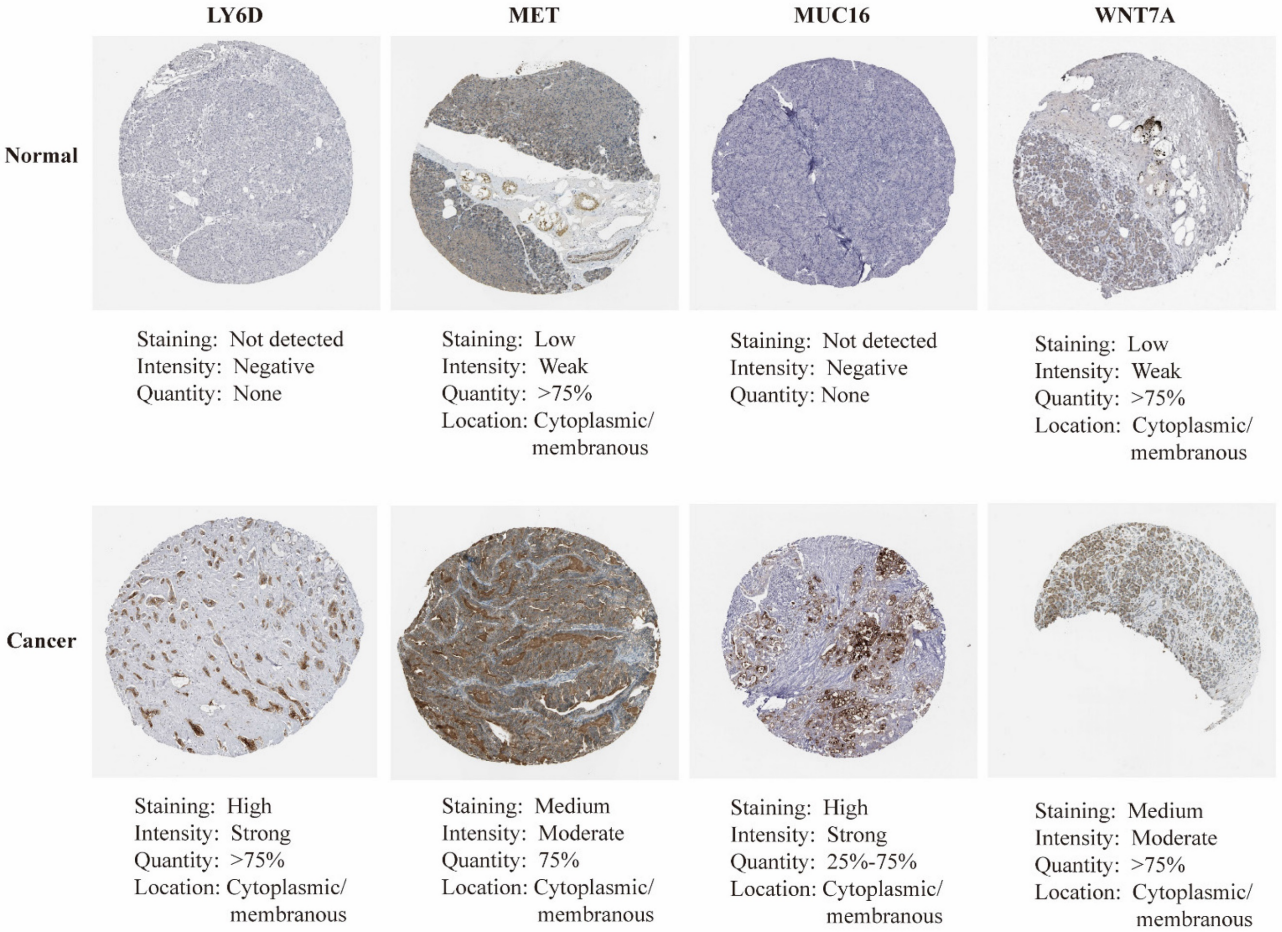
The immunohistochemical staining of key genes in PAAD and normal samples determined by HPA database.

mRNA expression of nine key genes was obtained from GEPIA database, and the protein expression of four key genes was collected from HPA database. mRNA expression of FAM83A, LY6D, MET, MUC16, MYEOV, and WNT7A were significantly elevated in cancer than healthy tissues (P<0.05) (Figure 11A). Survival curves revealed that high expression of these genes was significantly linked to bad outcome, except for MUC16 (P>0.05, Figure 11B). CDCC188, KHDRBS2, and SLC7A10 mRNA expression did not differ between cancer and healthy tissues (Figure S7A-C). In contrast, survival curves demonstrated that increased expression of these genes was significantly linked to bad outcome (Figure S7D-F). likewise, IHC staing affirmed that the protein expression patterns of LY6D, MET, MUC16, and WNT7A were increased in tumor sample compared to healthy samples (Figure 12).

## Discussions

Pancreatic adenocarcinoma, commonly known as the "king of cancers", is an aggressive disease with poor outcomes in the digestive system^13^. Symptoms are similar to those of other pancreaticobiliary diseases and are prone to misdiagnosis. Because the symptoms of the disease are insidious and progresses rapidly, it is already in a relatively late stage when it is discovered, so there is no very reasonable treatment plan when visiting a doctor^3^. At present, gene signatures of different molecular subtypes according to autophagy, metabolism, etc., have a significant impact on cancer^14^, ^15^. Individual risk assessment has good predictive value. Consequently, it is crucial to develop a molecular prediction model of PAAD to guide individualized treatment and predict prognosis.

Basement membranes (BMs) are is part of the extracellular matrix^4^. BMs Basement membranes protect tissues from damage and deformation, which also related to cell migration, differentiation and survival^16^-^18^. The variation of BM gene is associated with human diseases, reflecting the diversity and basic function of BM gene^19^. BM protein is also one of immunotherapy targets^20^ and the deletions of BM protein are one of the key factors of carcinogenesis^5^. There is no report on BM-related signatures in PAAD. We constructed a key prognostic risk score calculation model with eleven BMs (CCDC188, CSN1S1, FAM83A, KHDRBS2, LY6D, MET, MUC16, MYEOV, NIBAN3, SLC7A10, and WNT7A) in this study. The analysis showed that risk scores of BM-related signatures had excellent and independent prognostic significance. Additionally, the performance of the ROC curve was also excellent, which can well assess patients' prognoses and plan therapy.

Among these prognostic key genes, most of them have been reported to be related to the prognosis and disease progression of cancer patients. One gene of particular interest is CSN1S1, which has emerged as a distinctive and highly specific predictive marker, primarily associated with hepatocellular carcinoma^21^. FAM83A is a family member A gene with a sequence similarity of 83, which is a protein-coding gene. Extensive research has indicated a robust connection between FAM83A and the aggressive nature of tumor cells, making it a focal point for further investigation^22^-^24^. Next, KHDRBS2 has significant relation with the treatment and outcome of GBM patients^25^. Exploring the genetic terrain further, we have identified genes encoding proteins belonging to the lymphocyte antigen 6 (Ly6) superfamily. These genes are located on the long arm 24 (8q24) of human chromosome 8, in close proximity to the proto-oncogene c-Myc. These genes encode secreted proteins that exhibit wide distribution across various cell types, with a recurring pattern of overexpression in cancer tissues compared to their healthy counterparts^26^. LY6D is a prognostic marker of pancreatic adenocarcinoma and colorectal cancer^27^, ^28^. The main prevalent pathway of resistance to third-generation EGFR tyrosine kinase inhibitors (TKI) is MET amplification, which stimulates tumor cell STING, a major cancer immunogenicity driver^29^. SLC7A10 has emerged as a novel candidate molecular biomarker for cancer^30^. Moreover, ovarian, breast and lung cancers all exhibit abnormally high levels of MUC16^31^-^33^. MUC16 and its ligands are potential targets for treatment due to their dysregulation and functional involvement. MYEOV is largely upregulated and stimulates tumor development in several human cancers, such as gastric, colon, and non-small cell lung cancers (NSCLC) 34-36. WNT protein family is comprised of several cysteine-rich glycoproteins that are released, and 19 WNT genes have been found in the human genome 37. WNT signaling has been observed in several cancer forms, including but not restricted to colorectal, liver, and lung cancers^38^, ^39^. The above analysis is consistent with our results that BM genes are associated with tumor prognosis and progression. However, comprehensive analytical studies on PAAD and BMs are lacking. Therefore, more experimental evidence is needed to demonstrate the correlation between BMs and PAAD. Our research results unequivocally indicate that our proposed BM-associated signature displayed a predictive efficacy for PAAD prognosis that is either equivalent to or surpasses that of established models^40^-^42^. This finding served to reinforce the substantial importance of BM-related genes within the PAAD context.

Growing evidence suggests that immune cells in TME has a crucial function in disease progression^43^. Immunotherapy efficacy and overall survival significantly linked to TME components^44^. Our findings revealed that patients with low-risk score exhibited more immune cells infiltration like B, CD8+ T, and monocyte cells, which were more strongly associated with immune function, suggesting the important role of BM-associated genes in immune microenvironment. Immune checkpoint blockade (ICB) can increase the effect of antitumor immune responses^44^. Interestingly, BTLA, CD200, IDO1, LAG3, TNFSF14, etc., were overexpressed in the low-risk group, suggesting that low-risk patients may get better treatment outcomes in tumor immunotherapy. Also, we observed that low-risk patients showed higher StromalScore, ESTIMATEScore, and ImmuneScore. Various subtypes and immune scores can lead to various outcome and immunotherapy response^45^. Taken together, BM-related key prognostic genes can accurately and scientifically evaluate the prognosis of PAAD patients, which plays a crucial role in individualized immunotherapy for patients.

Recently, immunotherapy has played an important part as an approach to eliminate ICIs-based tumor cells in PAAD patients^46^. Nevertheless, the development of chemotherapeutic medication resistance in tumor patients caused great challenges to therapy and improvement of prognosis. Therefore, how to improve chemotherapy sensitivity has important clinical significance. Due to the existence of more neoantigens, there is proof that patients with a greater TMB respond better to immunotherapy 47. We found an association of TMB with risk score, lower TMB in the low-risk group by TMB analysis, The results indicate that these signatures can assess the efficacy of PAAD immunotherapy. Finally, we predicted some valuable chemotherapeutic agents for PAAD patients. High-risk patients are more sensitive to axitinib, camptothecin, nilotinib, and temisirolimus, while low-risk patients were more sensitive to bleomycin, bortezomib, dasatinib, erlotinib, paclitaxel, rapamycin, and sorafenib. Individualized chemotherapy combined with immunotherapy is necessary for patients having different risk scores. Above all, we predicted five small molecule drugs with potential therapeutic value for PAAD according to DEGs between two risk groups. First, Ro-90-7501 is an amyloid β42 (Aβ42) fibril assembly inhibitor that reduces Aβ42-induced cytotoxicity. Ro-90-7501 possesses significant radiosensitizing impacts on cervical cancer cells *in vitro*^48^. It could significantly delay tumor growth and significantly decreases tumor volume in mice^49^. Second, scriptaid is a potent histone deacetylase (HDAC) inhibitor, used in cancer research. Scriptaid strongly suppresses tumorigenesis in a xenograft mouse model^50^. Third, TG101348 is a potent and selective inhibitor of janus kinase 2 (JAK2). The therapeutic effect on mice with myeloproliferative disease induced by gene mutation was remarkable^51^. Fourth, XMD-892 is a unique, strong and highly selective dual inhibitor of BMK1/ERK5 (big mitogen activated protein kinase 1/extracellular-signal-regulated kinase) with potential antineoplastic activity. It significantly decreases the tumorigenesis via inducing CD8+ T cell antitumor effect^52^. Fifth, XMD-1150 could target one or more autophagy hub genes for accelerating autophagy modulation in cancer therapy. Here, we found five important small-molecule drugs for the treatment of PAAD that may help to find scientifically sound new therapeutic strategies for PAAD patients.

This research has some strengths and limitations. we report the value of integrating all BM-associated genes in the outcome, immune infiltration and drug prediction of PAAD for the first time. Then, the obtained genes can better predict the prognosis of PAAD patients by risk score. Lastly, five small molecule drugs with potential therapeutic value were predicted by related genes. A limitation of our research is that the mRNA and protein expression of cruical prognostic genes were verified only in the database. This needs to be further verified by basic experimental studies at all levels.

Overall, we developed and validated BM-associated genes having good performance in estimating PAAD outcome, immune cell infiltration, and chemotherapy response. Predicting potentially valuable small-molecule drugs through BM-related genes provides a new approach in drug therapy and is expected to enhance the outcome and quality of life of PAAD patients. BM-related genes may provide important reference for PAAD patients to obtain more scientific and effective individualized treatment plans, and can be used as auxiliary diagnosis and therapeutic tools for clinicians.

## Supplementary Material

Supplementary figures and tables.
