# Supplementary figures and images for: Identification of basement membrane-related signatures for estimating prognosis, immune infiltration landscape and drug candidates in pancreatic adenocarcinoma
Source: J Cancer. 2024 Jan 1;15(2):401–17. doi: 10.7150/jca.89665 (PMC10758037; doi:10.7150/jca.89665)

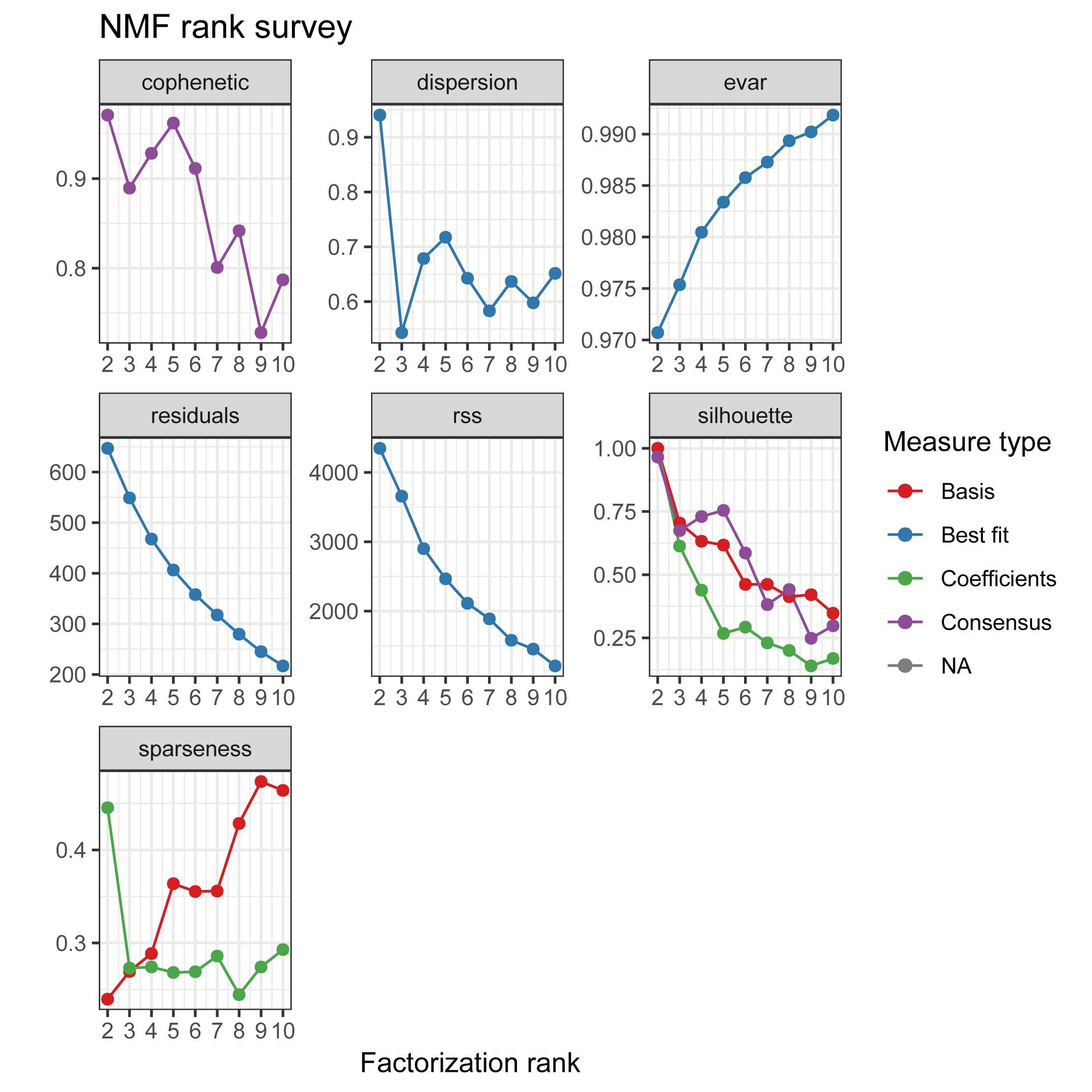

Supplement: Supplementary file 1 — Supplementary figures and tables. [file jcav15p0401s1.zip › Supplementary materials/FIGURE S1.tif]

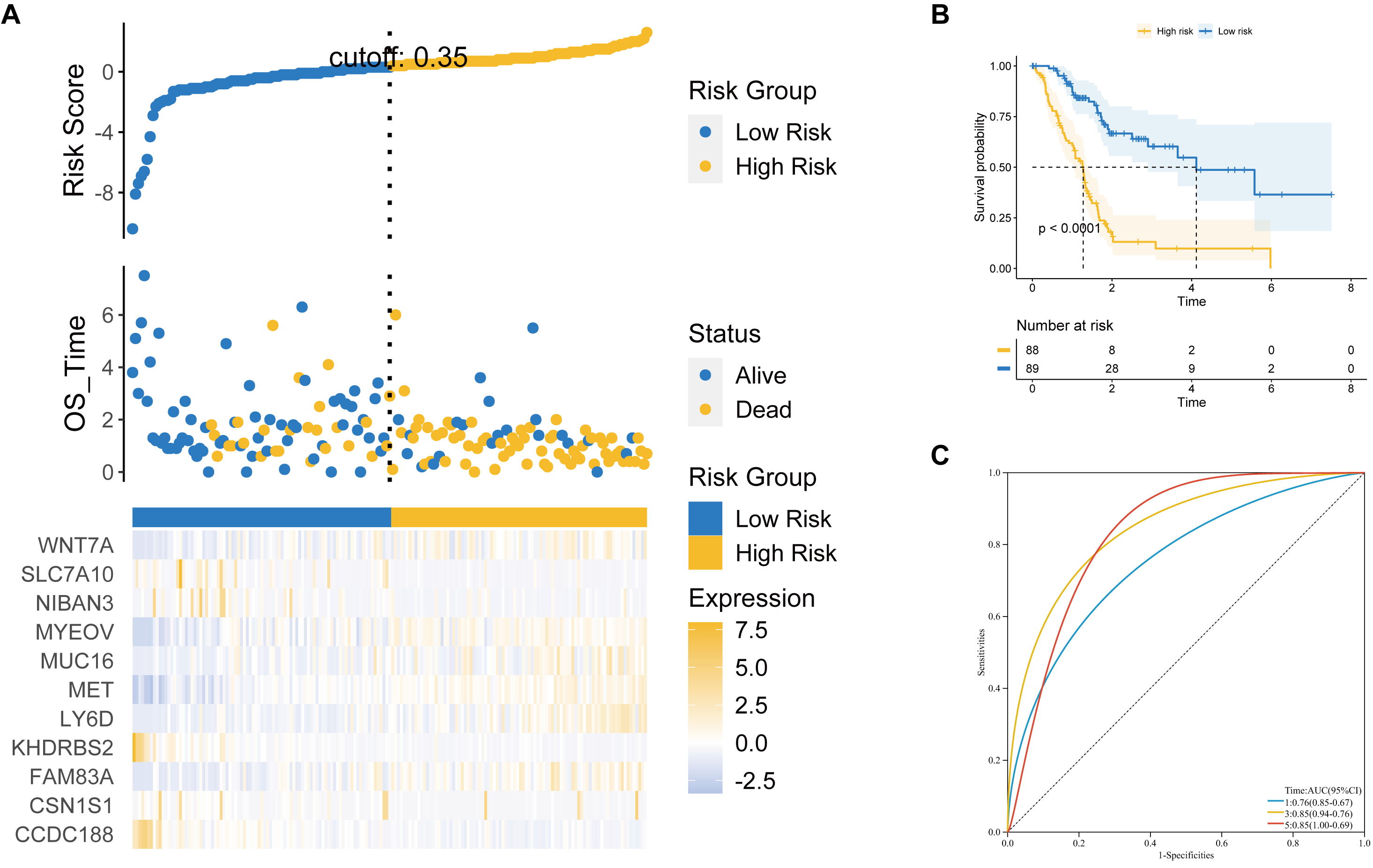

Supplement: Supplementary file 1 — Supplementary figures and tables. [file jcav15p0401s1.zip › Supplementary materials/FIGURE S2.tif]

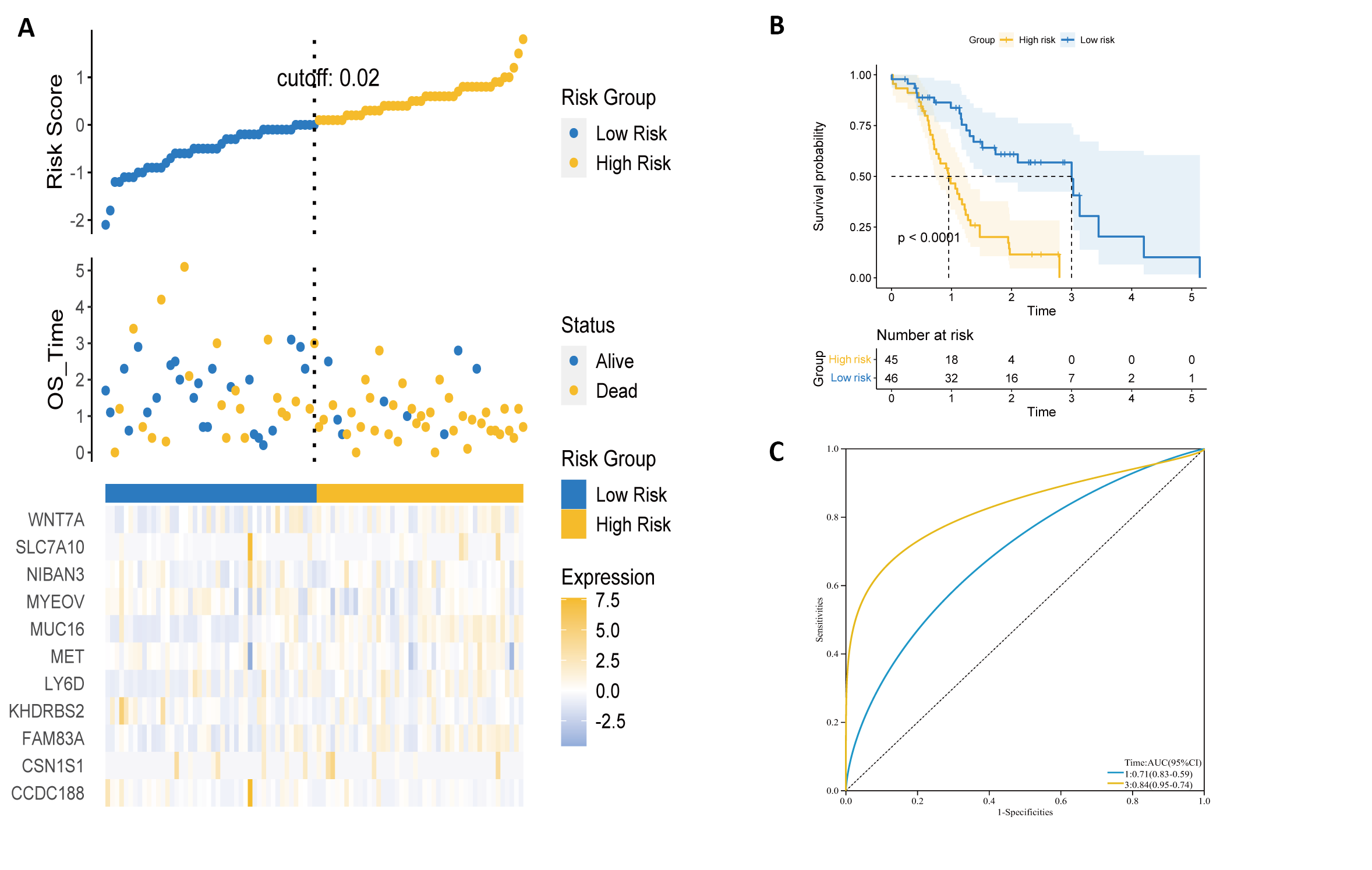

Supplement: Supplementary file 1 — Supplementary figures and tables. [file jcav15p0401s1.zip › Supplementary materials/FIGURE S3.tif]

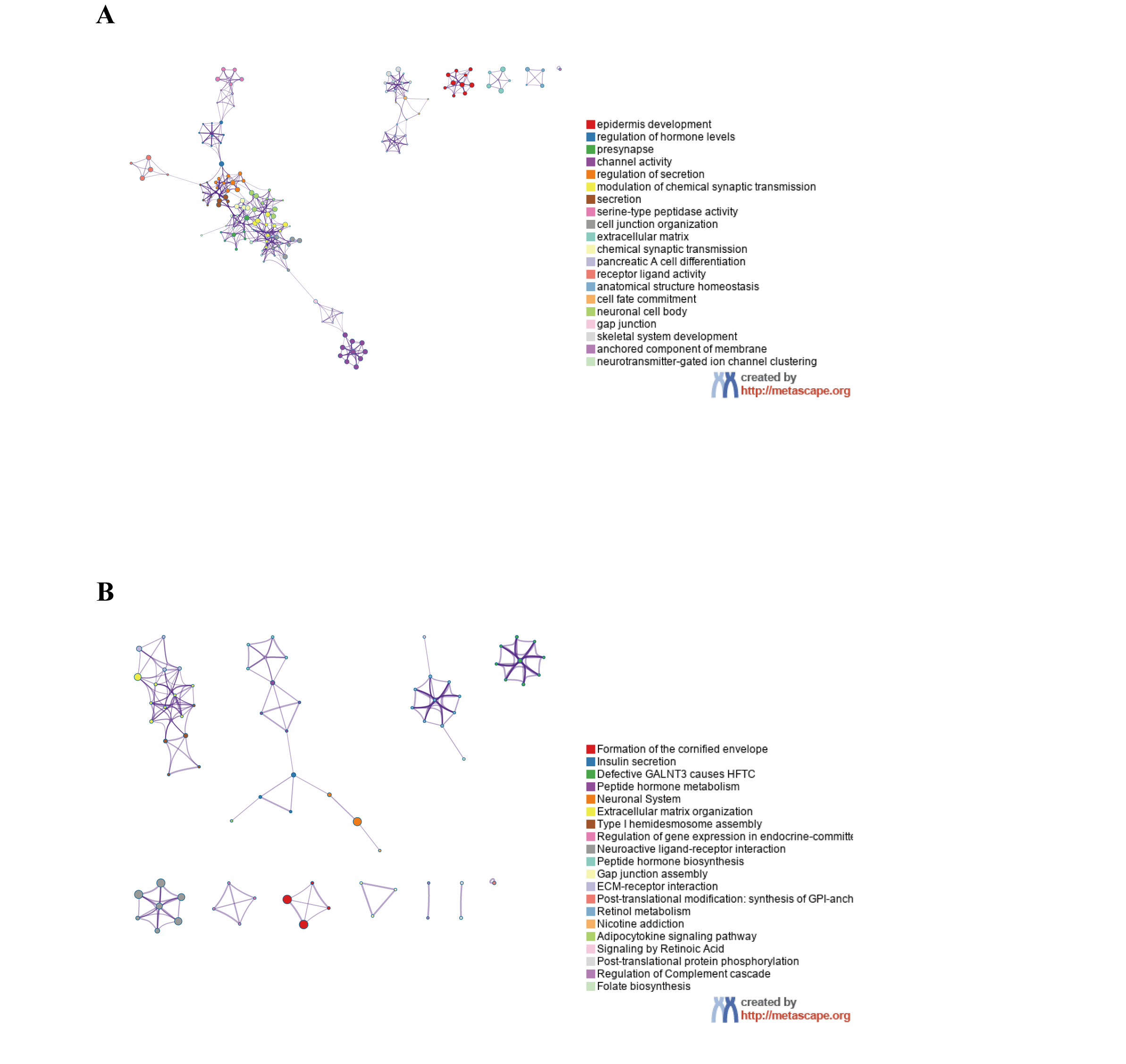

Supplement: Supplementary file 1 — Supplementary figures and tables. [file jcav15p0401s1.zip › Supplementary materials/FIGURE S4.tif]

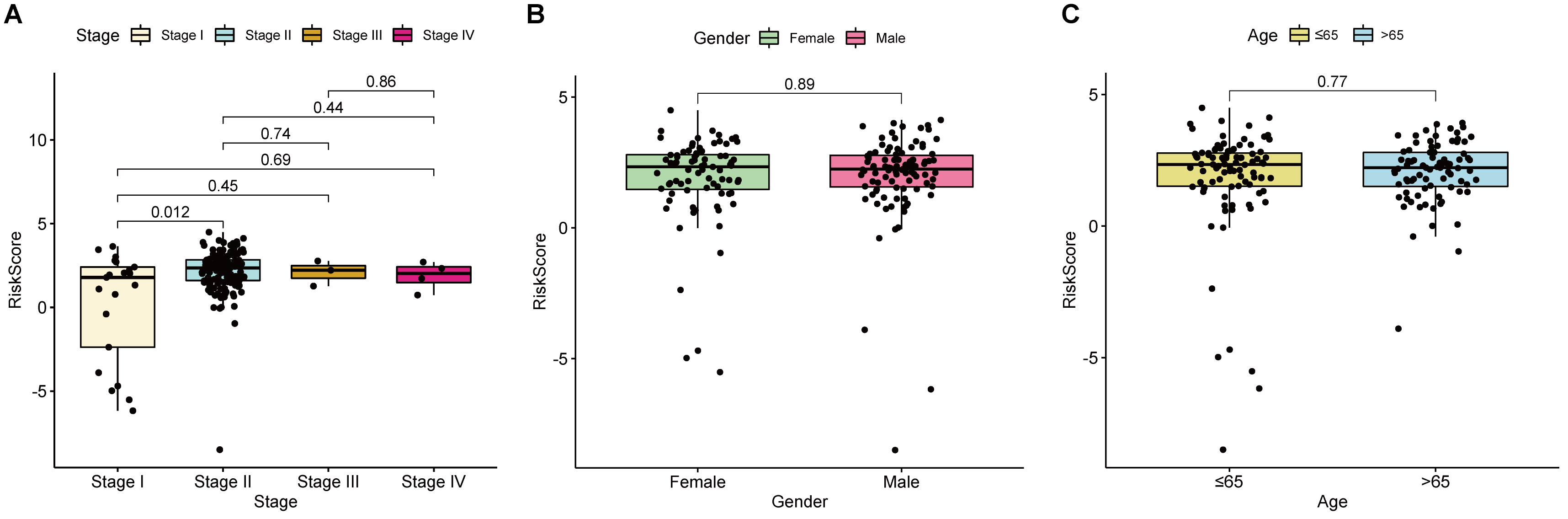

Supplement: Supplementary file 1 — Supplementary figures and tables. [file jcav15p0401s1.zip › Supplementary materials/FIGURE S5.tif]

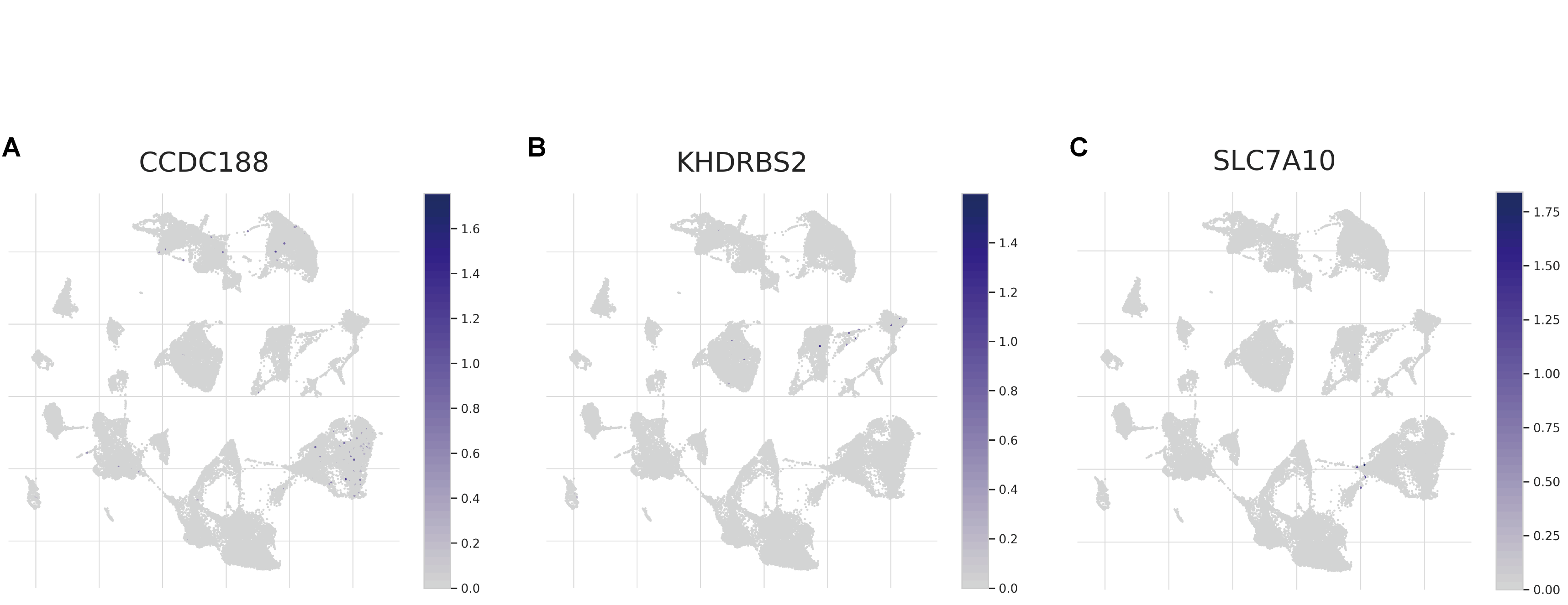

Supplement: Supplementary file 1 — Supplementary figures and tables. [file jcav15p0401s1.zip › Supplementary materials/FIGURE S6.tif]

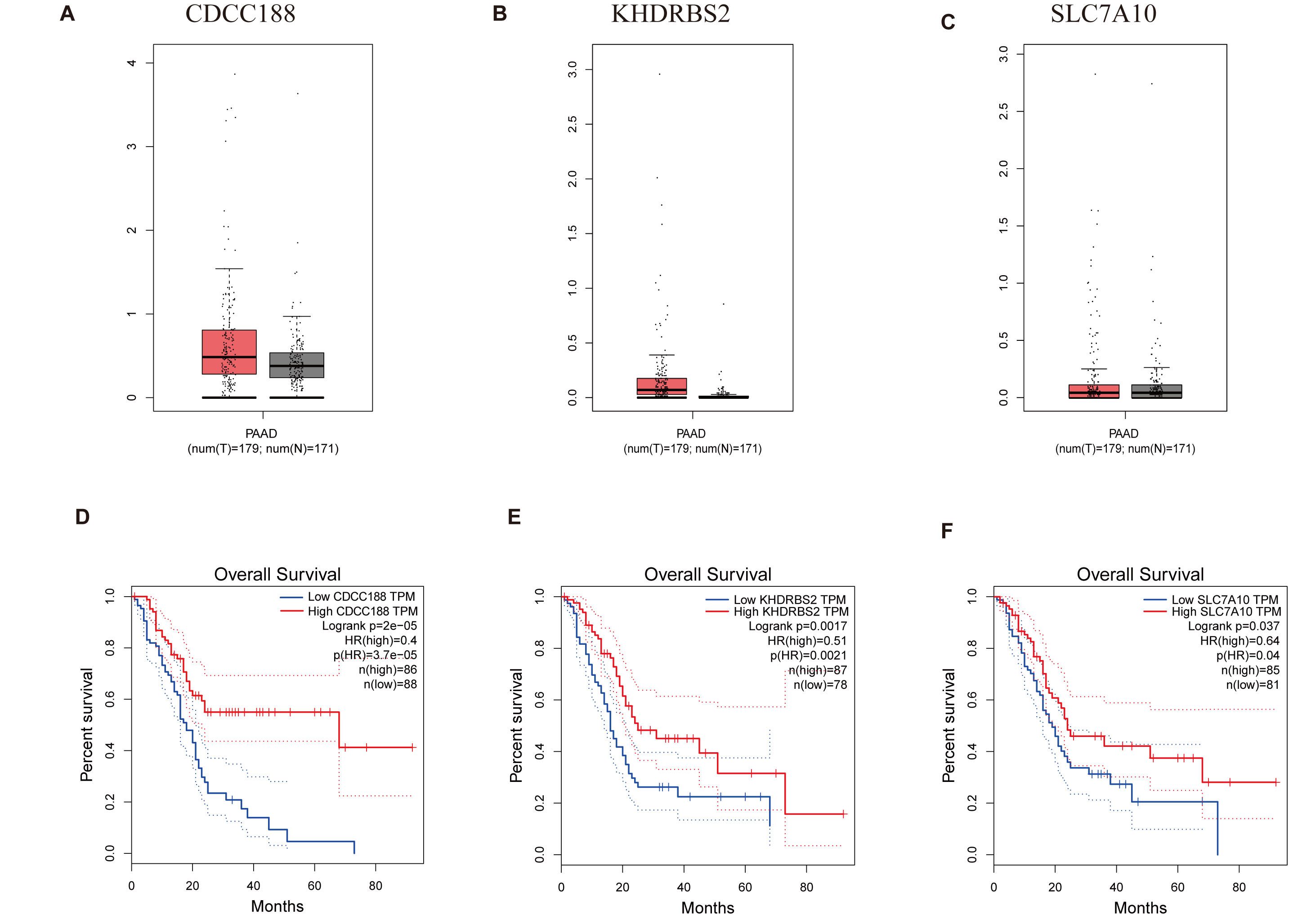

Supplement: Supplementary file 1 — Supplementary figures and tables. [file jcav15p0401s1.zip › Supplementary materials/FIGURE S7.tif]
